# Involvement of *dachshund* and *Distal-less* in distal pattern formation of the cricket leg during regeneration

**DOI:** 10.1038/srep08387

**Published:** 2015-02-11

**Authors:** Yoshiyasu Ishimaru, Taro Nakamura, Tetsuya Bando, Yuji Matsuoka, Hideyo Ohuchi, Sumihare Noji, Taro Mito

**Affiliations:** 1 Department of Life Systems, Institute of Technology and Science, The University of Tokushima Graduate School, 2-1 Minami-Jyosanjima-cho, Tokushima City, Tokushima, 770-8506, Japan; 2 Graduate School of Medicine, Dentistry and Pharmaceutical Sciences, Okayama University, 2-5-1 Shikata-cho, Kita-ku, Okayama City, Okayama, 700-8530, Japan; 3 Center for Collaboration among Agriculture, Industry and Commerce, The University of Tokushima, 2-24 Shinkura-cho, Tokushima City, Tokushima 770-8501, Japan

## Abstract

Cricket nymphs have the remarkable ability to regenerate a functional leg following amputation, indicating that the regenerating blastemal cells contain information for leg morphology. However, the molecular mechanisms that underlie regeneration of leg patterns remain poorly understood. Here, we analyzed phenotypes of the tibia and tarsus (three tarsomeres) obtained by knockdown with regeneration-dependent RNA interference (rdRNAi) against *Gryllus dachshund* (*Gb'dac*) and *Distal-less* (*Gb'Dll*). We found that depletion of *Gb'Dll* mRNA results in loss of the tarsal segments, while rdRNAi against *Gb'dac* shortens the tibia at the two most distal tarsomeres. These results indicate that *Gb'Dll* expression is indispensable for formation of the tarsus, while *Gb'dac* expression is necessary for elongation of the tibia and formation of the most proximal tarsomere. These findings demonstrate that mutual transcriptional regulation between the two is indispensable for formation of the tarsomeres, whereas *Gb'dac* is involved in determination of tibial size through interaction with *Gb'ds*/*Gb'ft*.

The principal differences between leg development and leg regeneration are in their initiation^1^. In the amputated leg, the starting point of regeneration consists of multiple differentiated tissues such as muscle, epidermis, peripheral nerve components, and various cells. Thus, leg regeneration relies on cell dedifferentiation in invertebrates^2^ as well as in vertebrates^1^. Following leg amputation, a blastema consisting of the dedifferentiated cells is formed at the amputated surface. How the blastema can redifferentiate to form the lost portion of the leg is a long-standing problem, and how regenerating cells have positional identity in the leg remains unknown.

We used the cricket *Gryllus bimaculatus*^3^ to elucidate the molecular mechanisms underlying development and regeneration of the leg. A cricket leg consists of six basic segments along the proximodistal (PD) axis: the coxa, trochanter, femur, tibia, tarsus, and claw. The cricket tarsus is subdivided into three tarsomeres^4^, which are not true segments as they lack the individual musculature seen in the other segments^5^. We observed that when the tibia of the cricket third-instar nymph is amputated at any level of the PD axis, the leg regenerates and recovers its allometric size and proper shape in the sixth instar, and the amputated leg is subsequently restored to almost normal adult leg size and shape^6^. Our previous work has shown that *Gryllus* *wingless* (*Gb'wg*) and *decapentaplegic* (*Gb'dpp*) are expressed in the ventral and dorsal sides of blastemal cells, respectively, while *Gryllus hedgehog* (*Gb'hh*) is expressed in the posterior side of the blastema, similar to that observed in the leg bud^4^,^6^ and in the *Drosophila* leg imaginal disc^7^.

In *Drosophila*, genes involved in establishing the PD axis of the leg have been identified and include *Distal-less* (*Dll*), which encodes a homeodomain transcription factor and is indispensable for the development of distal leg parts^8^,^9^, *dachshund* (*dac*), which encodes a transcriptional co-repressor and is required for the development of medial leg parts^10^,^11^, and *extradenticle* (*exd*) and *homothorax* (*hth*), which encode homeodomain transcription factors and together instruct proximal leg fates^12^,^13^,^14^,^15^,^16^. Rauskolb^17^ designated these four genes as the “leg gap genes” by analogy with embryonic segmentation, because the absence of *Dll*, *dac*, and *hth* functions results in deletion of distal, intermediate, and proximal leg segments, respectively^10^,^16^,^18^,^19^,^20^. The expression of these genes roughly corresponds to the regions of the leg affected by their absence and is related to the initial crude positional values along the PD axis of the holometabolous fly legs.

To examine whether *Dll*, *dac*, *exd* and *hth* function as gap genes in the legs of other species, we observed their expression patterns in the developing hemimetabolous cricket leg bud and found that these patterns are essentially the same as those observed in the fly leg^3^,^21^. These findings imply that the functions of the leg gap genes are conserved in the insect leg.

*Gryllus* *Dll* (*Gb'Dll*) is expressed in the distal domain that corresponds to the three tarsomeres, which are designated here as tarsal segment 1, 2, and 3 (Ta1, 2, and 3), and in the distal tibia; *dac* (*Gb'dac*) is expressed in the proximal domain of Ta1 and in the tibia^21^. Our previous studies demonstrated that *Gb'Dll* and *Gb'dac* are expressed in regenerating blastema after tibial amputation in cricket nymphs, similar to that observed in the cricket leg bud^22^,^23^.

To determine the functions of the leg gap genes in the cricket leg, we performed loss-of-function analyses using regeneration-dependent RNAi (rdRNAi) that occurs specifically in the amputated leg of cricket nymphs injected with double-strand RNA (dsRNA) for a target gene^24^. The functions of the leg gap genes are generally indispensable in many organs besides the leg, and therefore their knockout mutants tend to be lethal. To overcome this, we used an rdRNAi knockdown approach, the apparent effect of which could be restricted to the regenerating leg. Thus, we could examine gene functions during leg development in a leg-regeneration system.

Recently, we successfully applied rdRNAi to elucidate the functions of the Dachsous (*Gb'*Ds)/Fat (*Gb'*Ft) signaling pathway. We demonstrated that tibial size and shape along the PD axis in the regenerating cricket leg are regulated through the *Gb'*Ds/*Gb'*Ft signaling pathway^25^,^26^,^27^. Furthermore, classical transplantation experiments showed that, when two leg stumps with discontinuous positional values were grafted, intercalary regeneration restored the missing positional values^24^,^28^. Interestingly, intercalary regeneration was not observed in *Gb'Ds*/*Gb'Ft* rdRNAi legs, indicating that *Gb'*Ds/*Gb'*Ft signaling is also essential for specification of positional identity in regenerating tibia^25^. These findings clearly show that rdRNAi is useful for analyzing gene functions during leg regeneration.

Here, we analyzed the functions of *Gb'Dll* and *Gb'dac* in regeneration of the tarsus and tibia. We demonstrated that mutual transcriptional regulation exists between *Gb'Dll* and *Gb'dac* in the tarsal segments, leading to proper pattern formation of these segments. The short-tibia phenotypes obtained by rdRNAi against *Gb'dac* closely resemble those obtained by rdRNAi against *Gb'ds*/*Gb'ft*. In addition, intercalary regeneration did not occur in legs treated with rdRNAi against *Gb'dac*, as observed in legs treated with rdRNAi against *Gb'ds*/*Gb'ft*. These results indicated that cell proliferation along the PD axis in regenerating tibia depends on the expression of *Gb'dac* through interaction with *Gb'ds*/*Gb'ft*. Based on our results, we proposed molecular cascades functioning in leg regeneration.

## Results

### RNAi against *Gryllus* *Distal-less* and *dachshund* inhibits regeneration of the leg along the proximodistal axis

**Figure 1:**
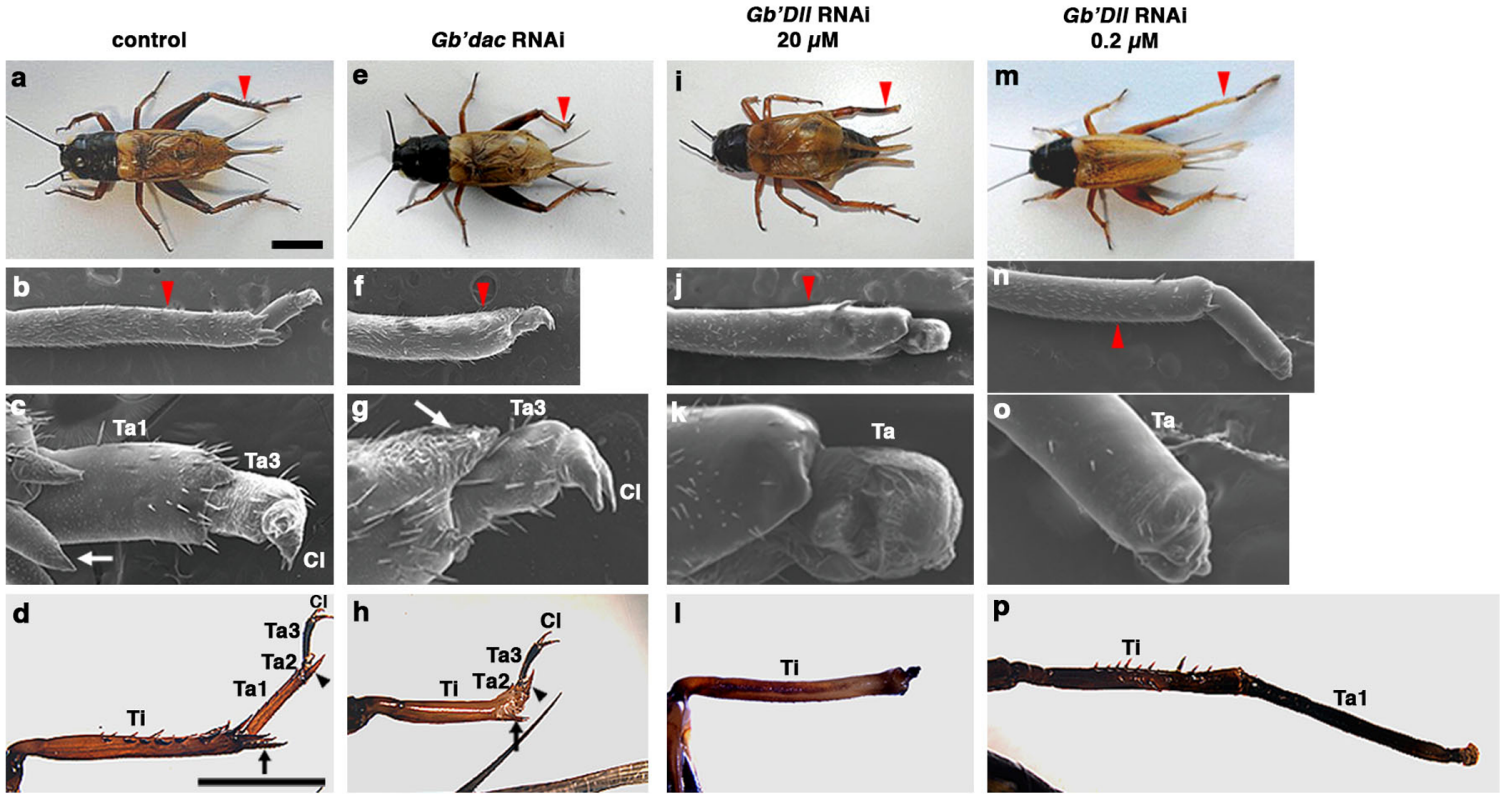
Leg phenotypes obtained by rdRNAi against *Gb'dac* and *Gb'Dll* during regeneration. (a) *DsRed2* rdRNAi control adult cricket with a normally regenerated right metathoracic (T3) leg. (b) Control regenerating leg of a fifth-instar nymph. (c) Higher magnification of panel b showing a control-regenerating tarsus. (d) Control regenerated leg of an adult. Normal tarsus consists of tarsal segments (Ta) 1, 2, and 3, and spurs and claws. (e) *Gb'dac* rdRNAi adult with a short regenerated T3 leg. (f) Regenerating leg of a fifth-instar nymph treated with *Gb'dac* rdRNAi. (g) Higher magnification of panel f showing a tarsus of *Gb'dac* rdRNAi regenerating leg. (h) Regenerated leg of a *Gb'dac* rdRNAi adult showing a normal Ta2 and 3, and claws but lacking Ta1 and having a short tibia. (i) *Gb'Dll* rdRNAi adult (injection of 20 μM *Gb'Dll* dsRNA). (j) Regenerating leg of a fifth-instar nymph treated with *Gb'Dll* (20 μM) rdRNAi. (k) Tarsus of regenerating *Gb'Dll* (20 μM) rdRNAi leg at higher magnification than shown in j. (l) Regenerated leg of a *Gb'Dll* (20 μM) rdRNAi adult. *Gb'Dll* (20 μM) rdRNAi caused extreme reduction of all tarsal structures. (m) A *Gb'Dll* (0.2 μM) rdRNAi adult. (n) Regenerating leg of a fifth-instar nymph treated with *Gb'Dll* (0.2 μM) rdRNAi. (o) Higher magnification of panel n showing a tarsus of *Gb'Dll* (0.2 μM) rdRNAi regenerating leg. (p) Regenerated leg of a *Gb'Dll* (0.2 μM) rdRNAi adult. A long Ta1 was formed. (b–c, f–g, j–k, and n–o) Scanning electron microscopy (SEM) of rdRNAi regenerating legs. Red arrowheads in (a–b), (e–f), (i–j), and (m–n) indicate the amputation site. Arrows indicate spurs of the tibiae in (c), (d), (g), and (h). Arrowheads indicate spurs of the tarsi in d and h. Scale bars; 5 mm in (a) and (d). Ta1–3, tarsal segments 1–3; Ti, tibia; Cl, claws.

We first examined whether rdRNAi occurs against *Gb'dac* or *Gb'Dll* during regeneration of the leg amputated at the tibia. We confirmed that dsRNA for *Gb'dac* or *Gb'Dll* injected into nymphs at the third instar without leg amputation (nymphal RNAi [nyRNAi])^24^ had no significant effect on legs (treated nymphs vs. adults, data not shown), suggesting that nyRNAi against *Gb'dac* or *Gb'Dll* does not affect normal leg growth. Next, we also confirmed that when a *DsRed2* dsRNA-injected control leg was amputated at the tibia (red arrowhead in Fig. 1a, b), the normal-looking shapes of the tarsus and claw were observed in a regenerating leg of a control fifth-instar nymph at 7 days post-amputation (dpa) (Fig. 1b, c), although these structures were slightly shorter than those of the contralateral leg. The regenerating tibia also recovered its length with the tibial spurs at the tibial end (Fig. 1b, c). Three tarsal segments with the tarsal spurs and claw were fully restored in the adult regenerated leg (Fig. 1d; *n* = 64/64). In contrast, when the right metathoracic legs of third-instar nymphs were amputated at the tibia immediately after injection of dsRNA for *Gb'dac* or *Gb'Dll*, the length of regenerated legs of treated adults was changed (Fig. 1e, i, m) relative to controls (Fig. 1a).

**Figure 2:**
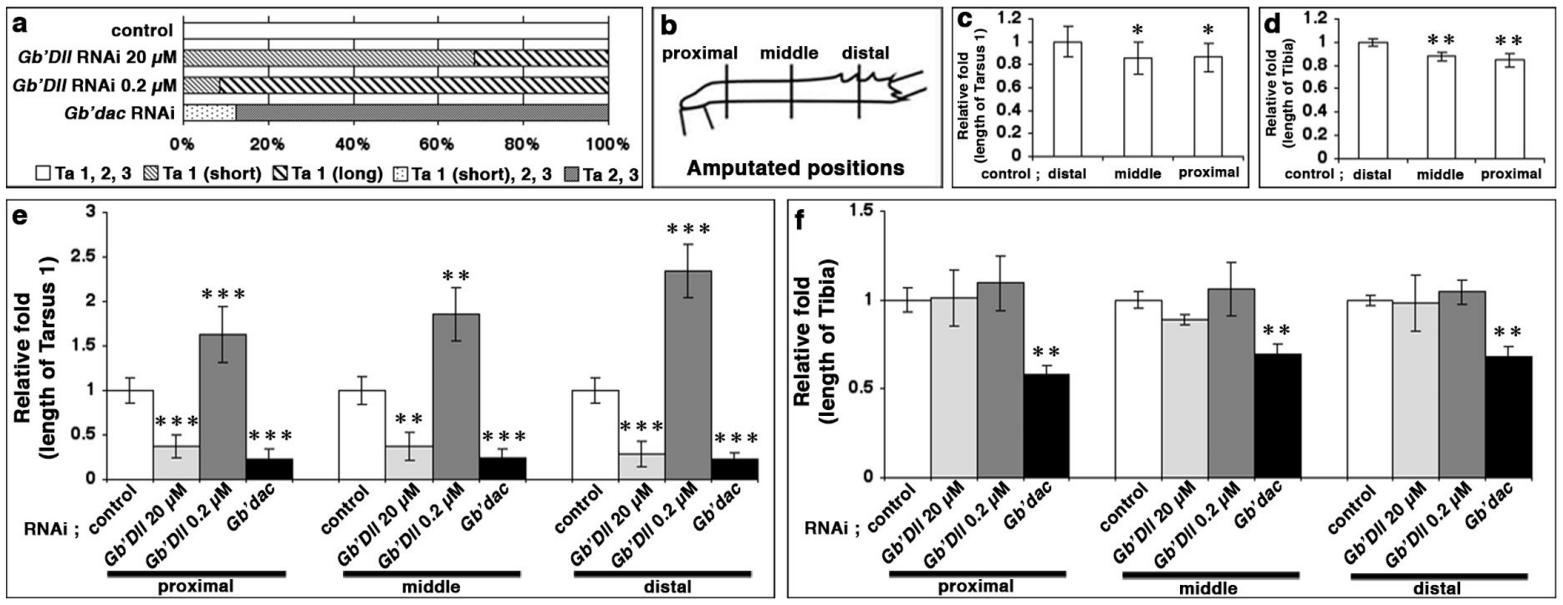
Effects of rdRNAi against *Gb'dac* and *Gb'Dll* on leg regeneration. (a) Bar graphs showing proportions of five different phenotypes of regenerated adult legs obtained by *DsRed2* rdRNAi, *Gb'dac* rdRNAi, *Gb'Dll* (20 μM) rdRNAi, and *Gb'Dll* (0.2 μM) rdRNAi. Open bar: phenotype of a regenerated leg with normal tarsal segment 1 (Ta1), 2 (Ta2), and 3 (Ta3). Thin striped bar: with only short Ta1. Thick striped bar: with only long Ta1. Dotted bar: with short Ta1 and normal Ta2 and Ta3. Gray bar: with Ta2 and Ta3. (b) A Schematic illustration showing the distal, middle, or proximal amputated positions in the tibia of third-instar nymphs. (c) Dependence of length of the regenerated Ta1 in control legs on amputation position, where relative length after distal amputation = 1. (d) Dependence of length of the regenerated tibia on amputation position. (e) Dependence of length of Ta1 treated with rdRNAi against control, *Gb'dac*, *Gb'Dll* (20 μM), and *Gb'Dll* (0.2 μM) on amputation position, where the average length of the control Ta1 = 1. (f) Dependence of length of the tibia treated with rdRNAi against control, *Gb'dac*, *Gb'Dll* (20 μM), and *Gb'Dll* (0.2 μM) on amputation position, where average length of the control tibia = 1. In panels (c–f), data are means ± SD. (Student's *t*-test; *P < 0.05, **P < 0.01, ***P < 0.001).

In the *Gb'dac* rdRNAi nymphs at 7 dpa, the regenerating leg became short and showed obvious defects in the tarsus and tibia (Fig. 1f). Ta1 failed to form, but no significant defects were observed in Ta2 and 3 or in claws (Fig. 1g). Furthermore, the regenerated tibia was shorter than the control tibia, and the tibial spurs appeared in the distal side (Fig. 1g). In the *Gb'dac* rdRNAi adult, Ta3 and claws were restored, whereas Ta1 was remarkably shortened (*n* = 8/64) or deleted (*n* = 56/64) (Figs. 1h and 2a). In addition to this structural change, the tibial spurs formed but tibia size along the PD axis was not fully restored (Fig. 1h; *n* = 61/64). These results indicate that the loss of *Gb'dac* function leads to defects in Ta1 and the tibia but does not affect the formation of Ta2 and 3 and the tibial decorations.

In *Gb'Dll* rdRNAi nymphs and adults, we found two distinct phenotypes in rdRNAi tarsi: either a short (Fig. 1j, l) or long (Fig. 1n, p) Ta1, depending on the dsRNA concentration of *Gb'Dll*. The regenerated tarsus of nymphs injected with dsRNA against *Gb'Dll* at a concentration of 20 μM became shorter (Fig. 1j), while at a lower concentration (0.2 μM), the regenerated tarsus became much longer than the normal tarsal segment (Fig. 1n). Interestingly, the claws and tarsal spurs were not clearly observed in either rdRNAi leg (Fig. 1k, o). In the *Gb'Dll* rdRNAi (20 μM) adult legs, Ta1 became short (Fig. 1l; *n* = 26/38), but the longer Ta1 was also observed in 31% of rdRNAi legs (Fig. 2a; *n* = 12/38). By contrast, approximately 91% of *Gb'Dll* rdRNAi (0.2 μM) legs showed the long-segment phenotype (Figs. 1p and 2a; *n* = 42/46). Ta2 and 3 and claws were entirely absent in *Gb'Dll* 20 and 0.2 μM rdRNAi legs. The regenerated tibiae of *Gb'Dll* rdRNAi legs achieved almost normal size (see Fig. 2f), whereas distal decorations, including tibial spurs, did not appear in *Gb'Dll* 20 μM rdRNAi legs (Fig. 1l). In *Gb'Dll* 0.2 μM rdRNAi legs, loss of tibial and tarsal spurs was an unstable phenotype (Fig. 1p). These results suggest that: (1) normal *Gb'Dll* expression is essential for formation of the tarsal segments; and (2) specification of the tarsal segments and formation of tibial spurs depend on the expression level of *Gb'Dll*.

To examine whether the leg phenotypes obtained by rdRNAi against *Gb'dac* or *Gb'Dll* depend on amputation position in the tibia, we measured the length of the regenerated adult tarsus and tibia after amputation at the proximal, middle, or distal level (Fig. 2b). We compared the length of Ta1 obtained by distal amputation (*n* = 11) with that obtained by middle (*n* = 8) or proximal (*n* = 12) amputation. Middle and proximal amputation reduced the length of Ta1 to approximately 86% (*t*-test; P < 0.05, *n* = 8) and 87% (*t*-test; P < 0.05, *n* = 12), respectively (Fig. 2c). The length of the regenerated tibia after middle and proximal amputation became short, to approximately 88% (*t*-test; P < 0.01) and 85% (*t*-test; P < 0.01) respectively, compared with that after distal amputation (Fig. 2d). These results indicated that amputation itself could reduce the size of the regenerated leg segment to 85% of adult length.

We then investigated the effects of amputation position on knockdown leg phenotype by rdRNAi against *Gb'dac* or *Gb'Dll* (dsRNA: 20 or 0.2 μM). In the case of *Gb'Dll* rdRNAi (20 μM), the relative ratios of length of Ta1 to the control were reduced to 37% (*t*-test; P < 0.001; *n* = 8), 37% (*t*-test; P < 0.01; *n* = 6), or 29% (*t*-test; P < 0.001; *n* = 12) after proximal, middle, or distal amputation (Fig. 2e). By contrast, in the case of *Gb'Dll* rdRNAi (0.2 μM), the relative ratios of Ta1 increased to approximately 163% (*t*-test; P < 0.001; *n* = 10), 186% (*t*-test; P < 0.01; *n* = 6), or 234% (*t*-test; P < 0.001; *n* = 6) after proximal, middle, or distal amputation, respectively (Fig. 2e). The relative ratio of tibial length in *Gb'Dll* rdRNAi was not significantly different from that of the control group for any amputation position (Fig. 2f). For *Gb'dac* rdRNAi, the relative ratios of Ta1 were 23% (*t*-test; P < 0.001; *n* = 8), 25% (*t*-test; P < 0.001; *n* = 6) and 22% (*t*-test; P < 0.001; *n* = 10) after proximal, middle, and distal amputation, respectively (Fig. 2e); the relative ratios of tibial length were reduced to about 58% (*t*-test; P < 0.01; *n* = 10), 69% (*t*-test; P < 0.01; *n* = 6), and 68% (*t*-test; P < 0.01; *n* = 14) after proximal, middle and distal amputation (Fig. 2f). One-way analysis of variance (ANOVA) showed no significant differences in the relative ratio of length of Ta1 between sample groups for different amputation positions (*Gb'Dll* rdRNAi 20 μM, P = 0.6927; *Gb'Dll* rdRNAi 0.2 μM, P = 0.0752; *Gb'dac* rdRNAi, P = 0.8403). The relative ratios of length of the tibial segment in *Gb'dac* rdRNAi also did not differ significantly between sample groups of amputation position (ANOVA; P = 0.1335). These results suggest that the distinct tarsal phenotypes observed by *Gb'Dll* rdRNAi (20 μM and 0.2 μM) are independent of tibia amputation position. We conclude that *Gb'dac* is involved in size determination of the tibia and Ta1, whereas *Gb'Dll* plays essential roles in the formation of the three tarsal segments during regeneration. It is interesting to note that size determination of Ta1 depends on the expression levels of both *Gb'dac* and *Gb'Dll*.

### Mutual transcriptional regulation between *Gb'dac* and *Gb'Dll* in regenerating tarsal segments

**Figure 3:**
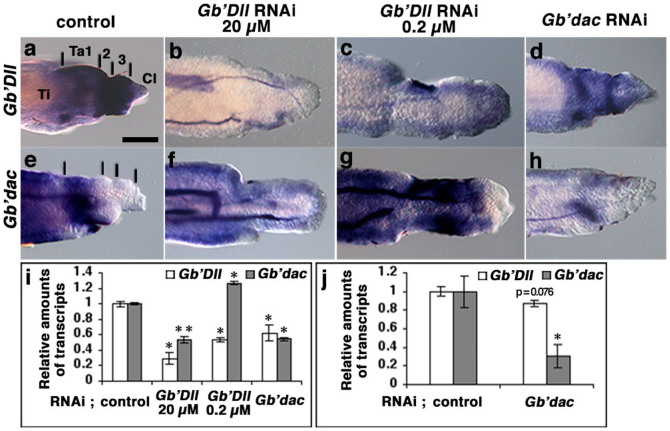
Effects of rdRNAi against *Gb'Dll* or *Gb'dac* on expression patterns of *Gb'dac* and *Gb'Dll* in regenerating tarsus. (a–h) Expression patterns of *Gb'Dll* and *Gb'dac* in control and rdRNAi regenerating tarsi at 5 dpa, obtained by whole-mount *in situ* hybridization. Expression patterns of *Gb'Dll* in regenerating tarsi treated with *DsRed2* rdRNAi (a), *Gb'Dll* (20 μM) rdRNAi (b), *Gb'Dll* (0.2 μM) rdRNAi (c), or *Gb'dac* rdRNAi (d). Vertical lines in panels a and e indicate borders of plausible tarsal segments 1, 2, and 3. (e–h) Expression patterns of *Gb'dac* in regenerating tarsi treated with *DsRed2* rdRNAi (e), *Gb'Dll* (20 μM) rdRNAi (f), *Gb'Dll* (0.2 μM) rdRNAi (g), or *Gb'dac* rdRNAi (h). (i) Relative ratios (control = 1) of *Gb'Dll* and *Gb'dac* mRNA in regenerating tarsi treated with *Gb'Dll* (20 μM) rdRNAi, *Gb'Dll* (0.2 μM) rdRNAi, or *Gb'dac* rdRNAi at 5 dpa. Relative ratios of *Gb'Dll* and *Gb'dac* are shown by white and grey bars, respectively. (j) Relative ratios (control = 1) of quantity of *Gb'Dll* and *Gb'dac* mRNA in the blastema at 2 dpa, treated with *Gb'dac* rdRNAi. Relative ratios of quantities of *Gb'Dll* and *Gb'dac* mRNA are shown by white and grey bars, respectively. In panels i and j, the data are means ± SD of three biological replicates. (Student's *t*-test; *P < 0.05, **P < 0.01). Scale bar in panel a = 100 μm. Ti, tibia; Cl, claws.

To elucidate expression patterns of *Gb'Dll* and *Gb'dac* during regeneration, we performed whole-mount *in situ* hybridization. Tracheal tubes shown in the *in situ* hybridization figures were artificially stained. In the *Gryllus* limb bud, after the major leg segments are established, both *Gb'dac* and *Gb'Dll* are expressed in the presumptive tibial segment and in a proximal area of the presumptive tarsal segment, whereas only *Gb'Dll* is expressed in the distal area of the presumptive tarsal segment^21^. Expression patterns of *Gb'Dll* and *Gb'dac* in regenerating legs are essentially similar to those in the limb bud. At 5 dpa, *Gb'Dll* expression was more intense in distal Ta2 and 3 than in Ta1 and a distal region of the tibia (Fig. 3a; *n* = 18), and *Gb'dac* was expressed in the proximal region of Ta1 and in the tibial segment, except for the most distal region of the tibia (Fig. 3e; *n* = 21). Expression of *Gb'Dll* was not detected in the claw. We speculate that intense expression of *Gb'Dll* may suppress expression of *Gb'dac* in Ta2 and 3, while weak expression of *Gb'Dll* in Ta1 might induce *Gb'dac* expression. To test this, we observed expression patterns of *Gb'dac* and *Gb'Dll* in regenerating leg with rdRNAi against *Gb'Dll* (20 or 0.2 μM) at 5 dpa. In the case of *Gb'Dll* rdRNAi (20 μM), *Gb'Dll* expression became significantly weaker than that of the control (Fig. 3b; *n* = 7/10), while *Gb'dac* expression was also substantially reduced in Ta 1 but persisted in regenerating tibia (Fig. 3f; *n* = 13/18). In contrast, in the case of *Gb'Dll* rdRNAi (0.2 μM), weak expression of *Gb'Dll* was present in the whole tarsus (Fig. 3c; *n* = 8/10), whereas the expression of *Gb'dac* became intense in the distal tarsus (Fig. 3g; *n* = 14/14). To confirm that the amounts of *Gb'dac* and *Gb'Dll* mRNA were decreased by rdRNAi, we performed qPCR and estimated the ratios of the amount of *Gb'dac* or *Gb'Dll* mRNA in comparison with the corresponding control (*n* = 11) at 5 dpa. The relative ratios of *Gb'Dll* mRNA were lowered to 29% (*t*-test; P < 0.05) and 53% (*t*-test; P < 0.05) in the rdRNAi tarsi against *Gb'Dll* with 20 μM dsRNA (*n* = 11) and 0.2 μM (*n* = 10), respectively (Fig. 3i), indicating that knockdown effects by rdRNAi depend on the concentration of *Gb'Dll* dsRNA. In the same case, the relative ratio of *Gb'dac* mRNA was reduced to 54% (*t*-test; P < 0.01) in rdRNAi tarsus against *Gb'Dll* (20 μM), while the relative ratio of *Gb'dac* increased to 127% (*t*-test; P < 0.05) in rdRNAi tarsus against *Gb'Dll* (0.2 μM) (Fig. 3i). These results demonstrate that increased *Gb'dac* expression due to lowering *Gb'Dll* expression levels may contribute to elongation of the regenerated Ta1.

Next, we found that *Gb'dac* rdRNAi reduced intense expression of *Gb'Dll* in Ta2 and 3 (Fig. 3d; *n* = 10/12), and reduced *Gb'dac* expression (Fig. 3h; *n* = 9/11) at 5 dpa. In the case of *Gb'dac* rdRNAi, the relative ratio of *Gb'Dll* mRNA decreased to 62% (*t*-test; P < 0.05; *n* = 12), while *Gb'dac* mRNA was depleted to 54% (*t*-test; P < 0.05) at 5 dpa (Fig. 3i). In blastemal cells at 2 dpa, *Gb'Dll* mRNA was reduced to 87% (*t*-test; P = 0.076; *n* = 10), concomitant with reduction of *Gb'dac* mRNA to 31% (*t*-test; P < 0.05) (Fig. 3j). Taken together, these results suggest that *Gb'dac* expression in Ta2 and 3 is suppressed by high expression of *Gb'Dll*, which was upregulated by *Gb'dac* in the early blastemal cells. Thus, we concluded that proteins of *Gb'Dll* and *Gb'dac* mutually regulate transcription and formation of the tarsal segments during regeneration.

### Effects of *Gb'Dll* and *Gb'dac* expression on cell proliferation in the regenerating leg

**Figure 4:**
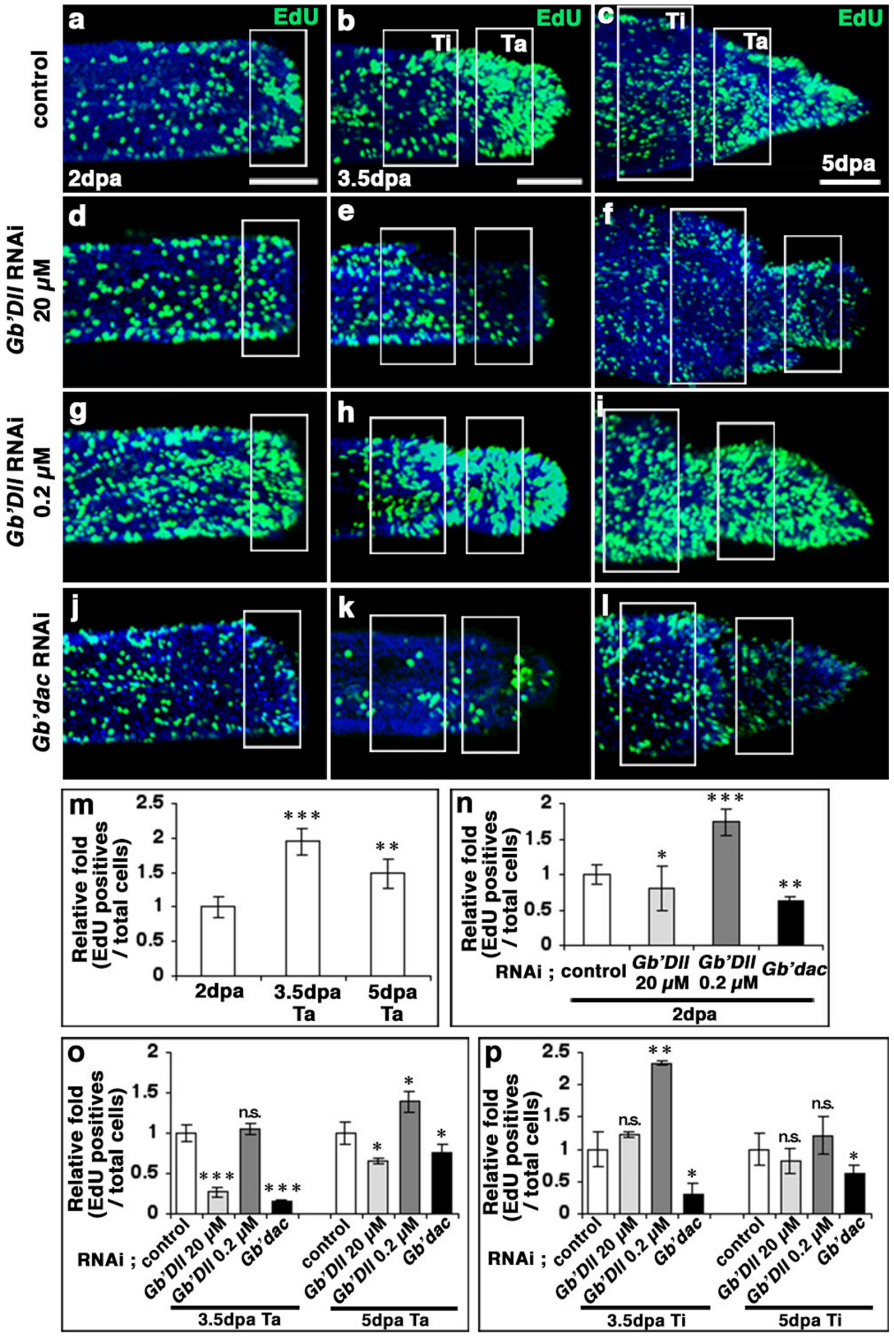
Effects of rdRNAi against *Gb'Dll* and *Gb'dac* on cell proliferation. (a–l) Localization of EdU-incorporated cells in blastemal regions at 2 dpa, and in regenerating tibiae and tarsi at 3.5 and 5 dpa for *DsRed2* rdRNAi (a–c), *Gb'Dll* rdRNAi (20 μM) (d–f), *Gb'Dll* rdRNAi (0.2 μM) (g–i), and *Gb'dac* rdRNAi (j–l) nymphs. EdU-positive and nuclei-merged cells are shown in green, and nuclei are shown in blue. Rectangles in panels (a), (d), (g), and (j) indicate blastemal regions at 2 dpa; rectangles in panels (b–c), (e–f), (h–i), and (k–l) indicate regenerating tibial regions (Ti) and tarsal regions (Ta). The panels in (a–l) show confocal z-stack images. (m) Relative fold changes in the numbers of EdU-positive cells in the blastemal region at 2 dpa and tarsus regions at 3.5 and 5 dpa in regenerating control legs are plotted, with the number in the blastemal region at 2 dpa set at 1. (n–p) Cell proliferation was analyzed quantitatively in blastemal regions at 2 dpa (n), in regenerating tarsal regions at 3.5 and 5 dpa (o), and in tibial regions at 3.5 and 5 dpa (p) in nymphs injected with *Gb'Dll* (20 μM), *Gb'Dll* (0.2 μM), and *Gb'dac* dsRNA. Relative fold changes in the numbers of EdU-positive cells in the blastemal region at 2 dpa, in regenerating tarsal regions at 3.5 and 5 dpa, and in tibial regions at 3.5 and 5 dpa in rdRNAi legs are plotted (numbers of EdU-positive cells, including controls, set at 1). In panels (m–p), the data are means ± SD. (Student's *t*-test; *P < 0.05, **P < 0.01, ***P < 0.001, n.s., not significant). Scale bars in (a–c) = 100 μm.

We observed that expression patterns of *Gb'Dll* and *Gb'dac* regulate the size of tarsal segments during regeneration. It is reasonable to consider that leg segment size depends on cell proliferation and/or cell death along the PD axis. Thus, we measured cell proliferation rate in regenerating control legs by EdU-incorporation assays^29^. The relative ratios of the number of EdU-positive cells to the total cells in the presumptive tarsus (Ta) at 3.5 dpa (Ta area in Fig. 4b; *n* = 5) and 5 dpa (Ta area in Fig. 4c; *n* = 7) increased to 195% (*t*-test; P < 0.001) and 149% (*t*-test; P < 0.01), respectively, compared to the relative ratios for the blastema at 2 dpa (rectangle in Fig. 4a; *n* = 6) (Fig. 4m). We next examined whether cell proliferation rates in regenerating legs were changed by *Gb'Dll* and *Gb'dac* rdRNAi. After injecting EdU into rdRNAi nymphs, we counted the number of EdU-positive cells at 2 dpa (rectangle in Fig. 4d, g, j). Interestingly, the number of EdU-positive cells decreased in blastemas treated with rdRNAi against *Gb'Dll* (20 μM, Fig. 4d; *n* = 6) or *Gb'dac* (Fig. 4j; *n* = 5), in comparison with the control (Fig. 4n). Conversely, the number of EdU-positive cells increased in blastemas treated with rdRNAi against *Gb'Dll* (0.2 μM) (Fig. 4g; *n* = 5) (Fig. 4n). We further examined the effect of *Gb'Dll* and *Gb'dac* rdRNAi on EdU incorporation rate into the tarsus (Ta) and tibia (Ti) at 3.5 and 5 dpa. In comparison with data for the control Ta, the relative ratios of the number of EdU-positive cells to total cells decreased to 27% (*t*-test; P < 0.001) at 3.5 dpa (Ta area in Fig. 4e; *n* = 5) and 65% (*t*-test; P < 0.05) at 5 dpa (Ta area in Fig. 4f; *n* = 5) for *Gb'Dll* rdRNAi (20 μM), and 15% (*t*-test; P < 0.001) at 3.5 dpa (Ta area in Fig. 4k; *n* = 5) and 76% (*t*-test; P < 0.05) at 5 dpa (Ta area in Fig. 4l; *n* = 6) for *Gb'dac* rdRNAi (Fig. 4o). By contrast, the number of EdU-positive cells in Ta treated with rdRNAi against *Gb'Dll* (0.2 μM) increased to 139% (*t*-test; P < 0.05) at 5 dpa (Ta area in Fig. 4i; *n* = 5), but there was no corresponding effect on Ta at 3.5 dpa (Ta area in Fig. 4h; *n* = 4). Whereas the numbers of EdU-positive cells decreased to 29% (*t*-test; P < 0.05) (Ti area in Fig. 4k) and 62% (*t*-test; P < 0.05) (Ti area in Fig. 4l) in tibiae treated with *Gb'dac* rdRNAi at 3.5 dpa and 5 dpa, respectively, compared with the control tibia (Fig. 4p). Next, we investigated whether apoptotic cell death occurs in regenerating legs. Only a few TUNEL-positive cells were detected in regenerating control legs, and their numbers did not increase in rdRNAi legs (data not shown). Taken together, these data suggest that an increase in proliferation rate in the presumptive tarsus at 3.5 dpa is primarily responsible for the tarsal growth, and that the proliferation is affected mainly by *Gb'dac* expression.

### A regulatory cascade of *Gb'Dll* and *Gb'dac* expression in regenerating legs

**Figure 5:**
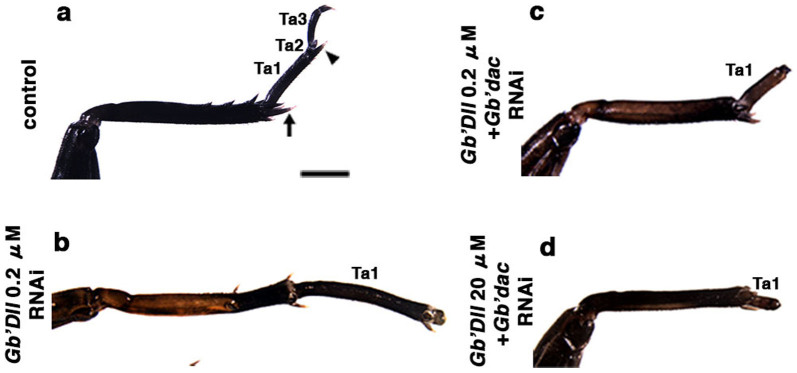
Effects of single or dual rdRNAi against *Gb'dac* and *Gb'Dll* on length of the tarsus. (a, b) Effect of single rdRNAi on phenotype of regenerating tarsus at the sixth instar. (c, d) Effect of dual rdRNAi on phenotype of regenerating tarsus at the sixth instar. (a) Tarsus in regenerating leg of a *DsRed2* rdRNAi nymph. (b) Distal elongated Ta1 of a nymph treated with *Gb'Dll* (0.2 μM) single rdRNAi. (c) Phenotype of Ta1 of a nymph treated with dual rdRNAi against *Gb'Dll* (0.2 μM) and *Gb'dac*. (d) Phenotype of Ta1 of a nymph treated with dual rdRNAi against *Gb'Dll* (20 μM) and *Gb'dac*. Ta1, Ta2, and Ta3 indicate tarsal segments 1, 2, and 3. Arrow and arrowhead (panel a) indicate spurs of the tibia and tarsus, respectively. Scale bar = 1 mm.

We found that elongated Ta1 obtained by rdRNAi against *Gb'Dll* (0.2 μM) formed due to an increase in cell proliferation induced by *Gb'dac* expression. Furthermore, we considered that weak expression of *Gb'Dll* induced by rdRNAi (0.2 μM) upregulates the expression of *Gb'dac*, which in turn activates cell proliferation. To confirm this, we analyzed phenotypes obtained by dual rdRNAi knockdown accomplished by simultaneous injection of two different dsRNAs. At the sixth instar, a control leg with normal tarsus was obtained by tibial amputation (Fig. 5a; *n* = 18/18). The long Ta1 obtained by single rdRNAi against *Gb'Dll* (0.2 μM) (Fig. 5b; *n* = 14/16) was changed to either normal or short length (Fig. 5c; *n* = 17/19) by dual rdRNAi against *Gb'dac*. In contrast, the short tarsus obtained by *Gb'Dll* rdRNAi (20 μM) showed no significant change by dual rdRNAi against *Gb'dac* (Fig. 5d; *n* = 11/11). These findings demonstrate that the expression of *Gb'dac* is induced by *Gb'Dll* in Ta1, but not in Ta2 and 3, where intense expression of *Gb'Dll* suppresses *Gb'dac* expression. It should be noted that the intense expression of *Gb'Dll* was induced by expression of *Gb'dac* in the blastema during the early stage of regeneration. Thus, these interactions between *Gb'Dll* and *Gb'dac* regulate the size of tarsal segments during regeneration.

### Regulation of *Gb'Dll* and *Gb'dac* on expression of distal patterning genes in the tarsus

**Figure 6:**
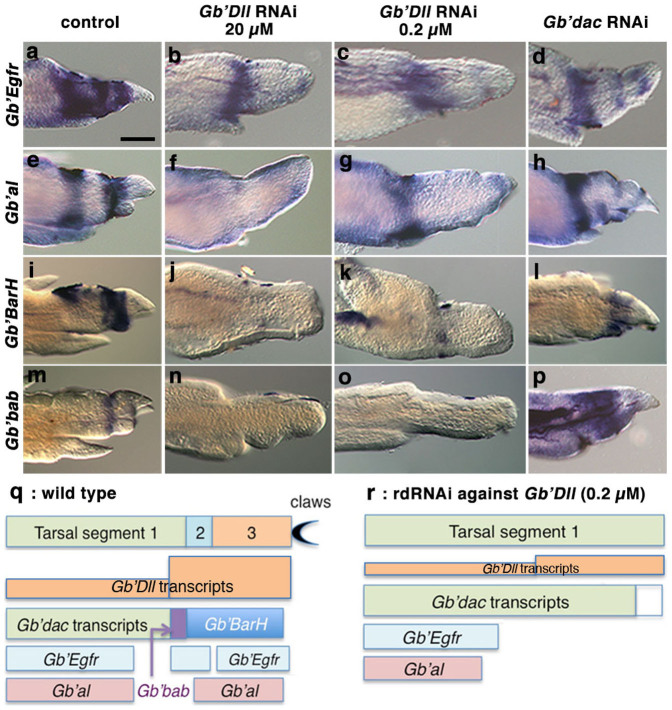
Effects of rdRNAi against *Gb'Dll* and *Gb'dac* on expression patterns of tarsal patterning genes, with schematic illustrations. (a–d) Expression patterns of *Gb'Egfr* in regenerating tarsi at 5 dpa for *DsRed2* rdRNAi (a), *Gb'Dll* rdRNAi (20 μM) (b), *Gb'Dll* rdRNAi (0.2 μM) (c), and *Gb'dac* rdRNAi (d) nymphs. (e–h) Expression patterns of *Gb'al* in regenerating tarsi at 5 dpa for *DsRed2* rdRNAi (e), *Gb'Dll* rdRNAi (20 μM) (f), *Gb'Dll* rdRNAi (0.2 μM) (g), and *Gb'dac* rdRNAi (h) nymphs. (i–l) Expression patterns of *Gb'BarH* in regenerating tarsi at 5 dpa for *DsRed2* rdRNAi (i), *Gb'Dll* rdRNAi (20 μM) (j), *Gb'Dll* rdRNAi (0.2 μM) (k), and *Gb'dac* rdRNAi (l) nymphs. (m–p) Expression patterns of *Gb'bab* in regenerating tarsi at 5 dpa for *DsRed2* rdRNAi (m), *Gb'Dll* rdRNAi (20 μM) (n), *Gb'Dll* rdRNAi (0.2 μM) (o), and *Gb'dac* rdRNAi (p) nymphs. (q) Schematic illustration of a plausible relationship between expression patterns of the tarsal patterning genes in a wild-type presumptive tarsus at 5 dpa and in tarsal segments 1–3. See details in the text. (r) Schematic illustration of a plausible relationship between tarsal segment-1-like structure and expression patterns of the tarsal patterning genes in a presumptive tarsus at 5 dpa observed in a nymph treated with rdRNAi against *Gb'Dll* (0.2 μM). See details in the text. Scale bar = 100 μm.

We found that *Gb'Dll* is involved in pattern formation of Ta2 and 3. In order to identify the target genes of *Gb'Dll* involved in patterning the distal tarsal segments, we observed the expression patterns of *Gryllus* orthologs of *Drosophila* tarsal-appendage-patterning genes such as *Epidermal growth factor receptor* (*Gb'Egfr*), *aristaless* (*Gb'al*), *BarH* (*Gb'BarH*), and *bric-a-brac* (*Gb'bab*). *Gb'Egfr* was expressed in the segmental boundaries in the tarsus and claw (Fig. 6a; *n* = 14), and *Gb'al* was expressed in Ta2 and 3 and in the tibia–tarsus boundary (Fig. 6e; *n* = 6). *Gb'BarH* was expressed broadly over Ta2 and 3 (Fig. 6i; *n* = 9), and *Gb'bab* expression occurred as a narrow circumferential ring in Ta2 (Fig. 6m; *n* = 10). We next examined the effects of *Gb'Dll*rdRNAi on the expression patterns of these genes. In regenerating legs treated with rdRNAi against *Gb'Dll* (either 20 μM or 0.2 μM), the expression of *Gb'Egfr* was significantly reduced in the two boundaries in the tarsus and claw, but persisted in the tibia–tarsus boundary (Fig. 6b, c; *n* = 12/14 or 10/12). Furthermore, the expression of *Gb'al* (Fig. 6f, g; *n* = 5/5 and 5/5), *Gb'BarH* (Fig. 6j, k; *n* = 7/7 and 7/7), and *Gb'bab* (Fig. 6n, o; *n* = 3/3 and 8/8) were significantly downregulated in rdRNAi legs against *Gb'Dll* (both 20 μM and 0.2 μM), while rdRNAi against *Gb'Dll* (0.2 μM) did not affect the expression of *Gb'al* in the tibia–tarsus boundary (Fig. 6g). These results suggest that depletion of *Gb'Dll* mRNA results in downregulation of these distal patterning genes, leading to lack of the distal tarsal segments. We also examined the effects of *Gb'dac* rdRNAi on the expression of distal patterning genes. The expression of *Gb'Egfr* (Fig. 6d; *n* = 13/16), *Gb'al* (Fig. 6h; *n* = 5/8), and *Gb'BarH* (Fig. 6l; *n* = 5/8) became weak in Ta2 and 3, but the expression of *Gb'Egfr* and *Gb'al* in the tibia–tarsus boundary was unchanged (Fig. 6d, h). In contrast, *Gb'bab* expression became broad in Ta1 (Fig. 6p; *n* = 6/8). Because depletion of *Gb'dac* mRNA reduces *Gb'Dll* expression in the distal tarsal segment, these results suggest that the expression of distal patterning genes is downregulated by lowered *Gb'Dll* mRNA levels, but that *Gb'bab* transcription in Ta1 is negatively regulated by *Gb'dac* expression.

Gene expression patterns in a wild type nymph are schematically illustrated in Figure 6q to show plausible correlations between expression patterns and tarsal segmentation (See also Fig. 8a). In the case of rdRNAi against *Gb'Dll* (0.2 μM), when *Gb'Dll* decreases to a certain threshold level, expression of *Gb'dac* is induced in the distal region, which then suppresses expression of the tarsal patterning genes (Fig. 6r). This change should induce formation of a long, Ta1-like structure.

### *Gb'dac* expression is regulated by expression of *Gryllus Dachsous* and *Fat* in the regenerating tibia

**Figure 7:**
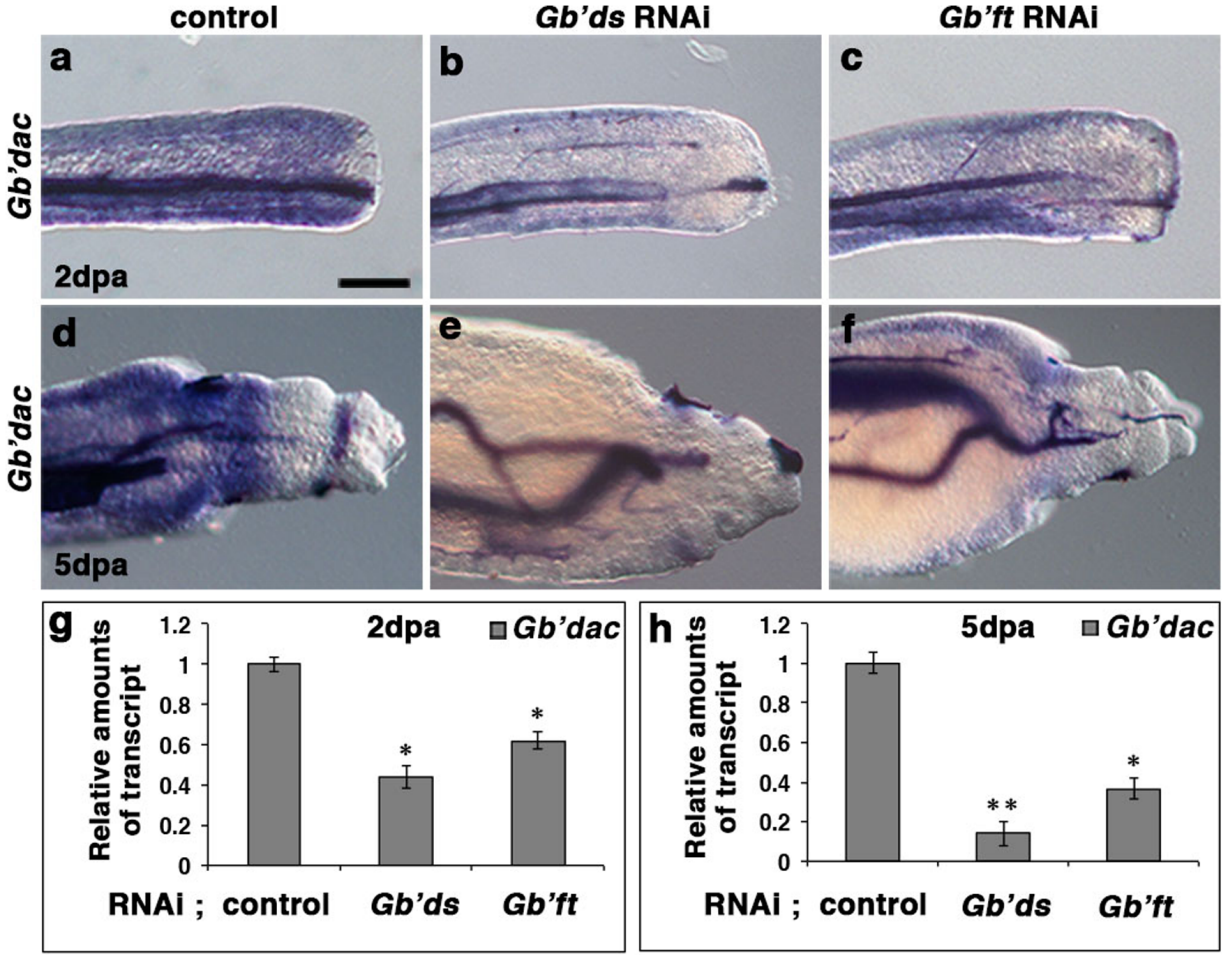
Effects of rdRNAi against *Gb'ds* and *Gb'ft* on expression patterns of *Gb'dac*. (a–c) Expression pattern of *Gb'dac* in regenerating legs at 2 dpa of *DsRed2* rdRNAi ((a); control), *Gb'ds* rdRNAi (b), and *Gb'ft* rdRNAi (c). (d–f) Expression pattern of *Gb'dac* in regenerating legs at 5 dpa of control (d), *Gb'ds* rdRNAi (e), and *Gb'ft* rdRNAi (f). (g) Quantities of *Gb'dac* transcripts at 2 dpa in blastemas treated with rdRNAi against *Gb'ds* or *Gb'ft*, relative to the control. (h) Quantities of *Gb'dac* transcripts at 5 dpa in regenerating tibiae and tarsi treated with rdRNAi against *Gb'ds* or *Gb'ft*, relative to the control. In panels g and h, data are means ± SD of three biological replicates. (Student's *t*-test; *P < 0.05, **P < 0.01). Scale bar = 100 μm.

We found that short-tibia phenotypes obtained by rdRNAi against *Gb'dac* resemble those obtained by rdRNAi against *Gb'ds*/*Gb'ft*; however, the thick phenotype induced by rdRNAi against *Gb'ds/Gb'ft* was not observed in the *Gb'dac* rdRNAi case. Therefore, we speculated that a relationship between *Gb'dac* and *Gb'ds*/*Gb'ft* is involved in regulating cell proliferation in the tibia. We hypothesized that expression of *Gb'dac* in the regenerating tibia may regulate cell proliferation through a *Gb'Ds*/*Gb'Ft* signaling pathway. To test this hypothesis, we analyzed expression of *Gb'dac* in regenerating legs treated with rdRNAi against *Gb'ds* or *Gb'ft*. In regenerating control legs at 2 dpa, *Gb'dac* expression was observed in the blastema and tibia but was not detected in the most distal tip of the blastema (Fig. 7a; *n* = 7). In regenerating legs treated with *Gb'ds* or *Gb'ft* rdRNAi, expression of *Gb'dac* was substantially reduced in the blastema at 2 dpa (Fig. 7b; *n* = 8/10 or Fig. 7c; *n* = 5/8). Interestingly, at 5 dpa the expression of *Gb'dac* was significantly reduced in Ta1 and whole tibia in *Gb'ds* rdRNAi legs (Fig. 7e; *n* = 16/20) relative to control legs (Fig. 7d; *n* = 10). The expression of *Gb'dac* was slightly decreased by *Gb'ft* rdRNAi at 5 dpa (Fig. 7f; *n* = 12/16). These changes in expression patterns were confirmed by qPCR (Fig. 7g, h). Relative quantities of *Gb'dac* transcripts in regenerating tibiae decreased due to rdRNAi against *Gb'ds*/*Gb'ft* at both 2 dpa (Fig. 7g) and 5 dpa (Fig. 7h). These results suggest that *Gb'dac* acts as a downstream factor of *Gb'Ds*/*Gb'Ft* signaling, which might control cell proliferation in the tibia.

## Discussion

**Figure 8:**
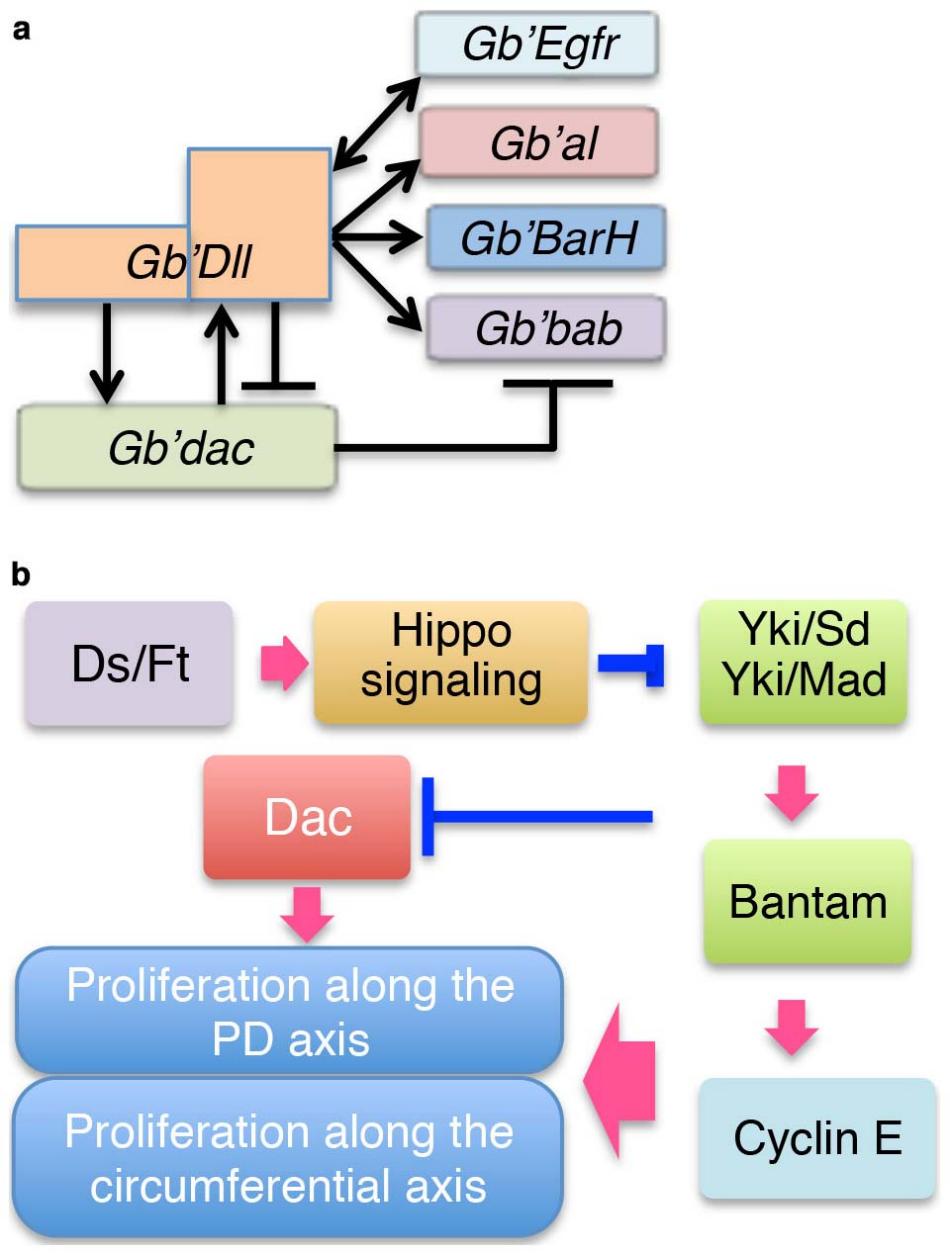
Schematic models of transcriptional regulation for regeneration of tarsal segments and for Ds/Ft signaling cascades for regulation of cell proliferation in regenerating tibia. (a) A schematic model for transcriptional regulation of tarsal repatterning genes to form the tarsal segments. After leg amputation, epidermal growth factor receptor (EGFR) signaling and *Gb'dac* induce a high level of *Gb'Dll* expression in the blastema. Then, low *Gb'Dll* expression induces *Gb'dac* expression in the proximal presumptive tarsal segment 1. In the distal presumptive tarsal segments 2 and 3, high *Gb'Dll* activity represses *Gb'dac* expression and induces the expression of *Gb'Egfr*, *Gb'al*, *Gb'BarH*, and *Gb'bab*, which establish the pattern of tarsal segments 2 and 3 along the proximodistal axis. *Gb'dac* expression represses *Gb'bab* expression in the distal tarsal segment 1. (b) A schematic illustration of the Ds/Ft signaling cascades in regeneration of the tibia to regulate tibial cell proliferation. Ds/Fat and Hippo signaling suppresses the activity of the Yki/Sd and Yki/Mad complexes, where Mad activity is regulated by Dpp signaling^36^. Both complexes control growth in part by regulating bantam and Cyclin E, which regulates cell proliferation. Expression of *Gb'dac* in the presumptive tibia is regulated by downstream genes of the Hippo signaling pathway. *Gb'*dac activated by formation of a complex with unknown factor(s) may induce cell proliferation.

Using an rdRNAi-knockdown approach against *Gb'Dll* and *Gb'dac*, we found that these genes are involved in regeneration of the tibia and tarsus after tibial amputation. Based on our experimental data, we propose a model for regulation of leg regeneration by *Gb'Dll* and *Gb'dac*, and discuss the following two points: (1) molecular cascades involved in tarsal segmentation during leg regeneration (Fig. 8a), and (2) regulation of cell proliferation in the regenerating tibia by *Gb'dac* (Fig. 8b).

When a cricket leg is amputated at the middle of the tibia, the whole tarsus and half of the tibia are lost. The regenerated blastema is formed in the distal region of the amputated leg, and blastemal cells proliferate and form the missing structures by intercalation between the most distal region and the remaining part of the leg. Since *Dll* is expressed by induction of Egfr signaling in the most distal region during limb development^30^ and regeneration^23^, *Gb'Dll* can be considered key for establishing the distal structures, especially the three tarsomeres. In addition, because *Gb'dac* is expressed in the tibial segment and in Ta1, it can also be considered to be involved in their formation. We illustrate the expression patterns of the genes studied here in the three tarsomeres, as shown in Fig. 6q, and their changes in the case of rdRNAi against *Gb'Dll* (0.2 μM) (Fig. 6r). A possible molecular cascade for establishing the three tarsal segments is illustrated in Fig. 8a. The cascade consist of two pathways, depending on the amount of *Gb'Dll* transcripts: when high expression of *Gb'Dll* is induced by signaling through *Gb'Egfr* in the most distal region of the blastema, *Gb'al*, *Gb'BarH*, and *Gb'bab* are expressed in the distal region (Figs. 6q and 8a), and formation of Ta2 and 3 may be regulated by their expression. In regions where *Gb'Dll* expression is low, expression of *Gb'dac* increases, represses *Gb'bab* expression, and induces formation of Ta1. This model is supported by the fact that when *Gb'Dll* mRNAs were depleted by rdRNAi, tarsal segmentation was abnormal (a long, Ta1-like structure was formed; Fig. 6r). Thus, we conclude that *Gb'*Dll acts as a negative or positive regulator for expression of *Gb'dac*, depending on its expression pattern, in formation of the tarsal segments.

In *Drosophila* leg development, *Dll* and *dac* act as patterning genes, specifying distal and proximal domains, respectively, along the PD axis^31^. In *Drosophila* *Dll* mutants, stronger allelic combinations produce loss of all tarsal segments^19^,^32^. In *Drosophila* leg imaginal discs, *Dll* is a direct activator of *dac* during early stages; but at late stages, *Bar* expression induced by *Dll* and EGFR signaling mediates *dac* repression directly by binding to multiple homeodomain binding sites^33^. In *Drosophila*, *bab* is required for proper folding of the leg imaginal disc^5^,^34^. These developmental roles of *Dll* and *dac* are essentially similar to those of *Gb'Dll* and *Gb'dac* in regenerating blastema in the *Gryllus* leg. Thus, we conclude that the leg-regeneration process recapitulates the developmental process, which may be conserved in the insect leg, supporting the concept of “distalization” (reviews^1^,^35^) in mechanisms of regeneration.

During the establishment of PD patterning in leg regeneration, cell proliferation appears to be promoted in the presumptive tibia in addition to the regenerating tarsus. Here, we focus on regeneration of the tibia. We found that (1) regenerated tibia treated with rdRNAi against *Gb'dac* became short, similar to the short-tibia phenotype obtained by rdRNAi against *Gb'ds*/*Gb'ft*^25^; (2) no intercalary regeneration occurs in the case of rdRNAi against *Gb'dac*, as observed in the case of rdRNAi against *Gb'ds*/*Gb'ft* (see supplemental section); (3) *Gb'dac* expression during leg regeneration overlaps with the expression domain of both *Gb'ds* and *Gb'ft* in the tibia; and (4) *Gb'dac* expression is positively regulated by *Gb'ds*/*Gb'ft* expression. Based on these results, we proposed a possible signaling cascade in tibial regeneration (Fig. 8b). Previous work showed that rdRNAi against *Gb'ds* and *Gb'ft* induces formation of a short and thick tibia with the normal short distal structures, including spines and spurs^25^. It is noteworthy that tibia treated with rdRNAi against *Gb'ds*/*Gb'ft* became short, despite the fact that cell proliferation was accelerated^26^. This shortening of the tibia is due to rearrangement of positional values in the amputated tibia. On the other hand, the thickening may be caused by cell proliferation that is probably promoted by cyclin E through inactivation of the Hippo/Warts signaling pathway, including *Gb'*Yokie (*Gb'*Yki)/*Gb*'Scalloped (*Gb'*Sd) or *Gb'*Yki/*Gb'*Mothers against dpp (*Gb*'Mad), and *Gb'*Bantam (Fig. 8b)^36^. Since the short-tibia phenotype found by knockdown of *Gb'dac* expression is not associated with tibia thickness, a signaling cascade involving *Gb'dac* should be different from that involving cyclin E (Fig. 8b). We have so far been unable to establish how *Gb'dac* expression is regulated by *Gb'Ds*/*Gb'Ft* signaling. However, we can conclude that *Gb'dac* expression affects tibial cell proliferation and helps to determine the size of the regenerating tibia along the PD axis, through the *Gb'Ds*/*Gb'Ft* signaling network.

## Methods

### Animals

All adult and nymph two-spotted crickets, *Gryllus bimaculatus*, were reared under standard conditions^6^,^23^,^24^.

### Regeneration-dependent RNAi (rdRNAi)

Preparation of double-stranded RNAs (dsRNAs) for *Gb'Dll*, *Gb'dac*, *Gb'ds*, and *Gb'ft*, and the rdRNAi method, were described previously^23^,^24^,^25^. After injection of dsRNAs into the abdomen of third-instar nymphs, their tibiae were amputated at the distal position between the second and third spines. Thus, this amputation removed 30% of the distal part of the tibia. The concentration of dsRNA for each gene was 20 μM, except for *Gb'Dll* injected at a lower dose (0.2 μM). The regeneration processes of RNAi nymph legs were observed in comparison with the negative control injected with dsRNA for *DsRed2*. *DsRed2* dsRNA was prepared as previously described^37^. Dual RNAi is performed by injecting a dsRNA mixture for two target genes, *Gb'dac* and *Gb'Dll* (20 or 0.2 μM). The final concentration of each dsRNA was adjusted to 20 μM (or 0.2 μM for lower-concentration *Gb'Dll*).

### Scanning electron microscopy (SEM)

The cricket rdRNAi legs were fixed in 4% paraformaldehyde and 4% glutaraldehyde in PBS overnight and were then washed three times in PBS for 15 min at room temperature. The rdRNAi legs were processed through an alcohol series (20, 40, and 60% ethanol in PBT; 80% ethanol in water; 45% ethanol and 45% *tert*-butyl alcohol in water; each for 40 min) and in 100% *tert*-butyl alcohol (>30°C) for 1 h. After substituting fresh 100% *tert*-butyl alcohol, the rdRNAi legs were frozen at 4°C, freeze dried (Hitachi ES-2030 dryer), and sputtered for 300 s with a 15-nm platinum coat (Hitachi ES-1020). The prepared samples were examined with an emission scanning electron microscope (Hitachi S-4700).

### Transplantation experiments for nymphal legs

A transplantation experiment for normal intercalary regeneration was performed as described previously^24^. Briefly, the experiments were performed with nymphs treated with *Gb'dac* rdRNAi. We used the amputated metathoracic leg stump as a host and the amputated mesothoracic distal part as a graft. To connect the legs, the mesothoracic graft was inserted into the metathoracic leg stump (see Supplementary Fig. S1a).

### Whole-mount *in situ* hybridization

Samples of regenerating legs were prepared and whole-mount *in situ* hybridization was performed as described previously^4^,^6^. A digoxigenin (DIG)-labeled antisense RNA probe for *Gb'Dll*, *Gb'dac*, *Gb'Egfr*, *Gb'al*, *Gb'BarH*, or *Gb'bab* was used for whole-mount *in situ* hybridization^23^.

### Cell proliferation assay

A cell proliferation assay was carried out using the Click-iT EdU Alexa Fluor 488 Imaging Kit (Invitrogen)^29^. In brief, EdU solution was injected into the abdomens of nymphs at the appropriate analysis stage, and regenerating legs were fixed 4 h after EdU injection^25^. Hoechst 33342 was used for nuclei staining.

### Quantitative PCR (qPCR)

Total RNA was extracted from the blastemal regions at 2 dpa or from regenerating tibiae and tarsi at 5 dpa of *DsRed2* (control), *Gb'dac*, *Gb'Dll* (20 or 0.2 μM), *Gb'ds*, and *Gb'ft* rdRNAi metathoracic (T3) legs. Left and right T3 legs from each individual were used for sampling the blastema or regenerating tibiae and tarsi. qPCR assays were performed in triplicate biological samples. In each assay, 11 nymphs at 2 dpa and 10 nymphs at 5 dpa were used for control legs; 12 nymphs at 2 dpa and 10 nymphs at 5 dpa were used for *Gb'dac* rdRNAi; 11 nymphs (5 dpa) were used for *Gb'Dll* 20 μM rdRNAi legs; 10 nymphs (5 dpa) were used for *Gb'Dll* 0.2 μM rdRNAi legs; 10 nymphs each (2 and 5 dpa) were used for *Gb'ds* and *Gb'ft* rdRNAi legs. The ABI 7900 Real-Time PCR System (Applied Biosystems) was used for qPCR as described previously^23^. The qPCR primer sequences were as follows: (forward and reverse, 5′ to 3′): *Gb'dac*, AACTACTCGGGGCTCGACCT and TCTTGACTTCCGCTCCATCTC; *Gb'Dll*, ACGGCAAGGGCAAGAAGA and AGTACTGCGTCCGCTGGAA. We used *Gb'β-actin* as an internal control^23^,^25^.

## Author Contributions

Y.I., T.N., S.N. and T.M. designed the work. Y.I. and T.N. performed all experiments and contributed equally to the work. Y.I., T.N., T.B., Y.M., H.O., S.N. and T.M. analyzed the data. Y.I., T.N., S.N. and T.M. prepared all figures and wrote the main manuscript text. All co-authors contributed in the form of discussion and critical comments.

## Supplementary Material

Supplementary Information Supplementary Information
