# Supplementary material for: Involvement of dachshund and Distal-less in distal pattern formation of the cricket leg during regeneration
Source: Sci Rep. 2015 Feb 11;5:8387. doi: 10.1038/srep08387 (PMC4323655; doi:10.1038/srep08387)
Supplement: Supplementary Information [file srep08387-s1.doc]

**Supplementary information**

Involvement of *dachshund* and *Distal-less* in distal pattern formation of the cricket leg during regeneration

Yoshiyasu Ishimaru^1^, Taro Nakamura^1#^, Tetsuya Bando^2^, Yuji Matsuoka^1^,

Hideyo Ohuchi^2^, Sumihare Noji^3^, and Taro Mito^1^*

^1^Department of Life Systems, Institute of Technology and Science, The University of Tokushima Graduate School, 2-1 Minami-Jyosanjima-cho, Tokushima City, Tokushima, 770-8506, Japan

^2^Graduate School of Medicine, Dentistry and Pharmaceutical Sciences, Okayama University, 2-5-1 Shikata-cho, Kita-ku, Okayama City, Okayama, 700-8530, Japan

^3^Center for Collaboration among Agriculture, Industry and Commerce, The University of Tokushima, 2-24 Shinkur-cho, Tokushima City, Tokushima 770-8501, Japan

^#^Present address: Department of Organismic and Evolutionary Biology, Harvard University, 16 Divinity Avenue, BioLabs 4109, Cambridge, MA 02138, USA

*Author for correspondence: mito@bio.tokushima-u.ac.jp

Running title: Roles of *dac* and *Dll* in tarsus regeneration

**Effects of rdRNAi against *Gb’dac* on intercalary regeneration in the tibia**

To examine the roles of *Gb’dac* in intercalary regeneration, we performed transplantation experiments by grafting an amputated mesothoracic leg (T2) as a donor to a metathoracic leg (T3) as a host in the same nymph (Supplementary Fig. S1a). Normal intercalary regeneration occurs in the control leg (upper panel in Supplementary Fig. S1b; *n =* 11/11), while normal intercalary regeneration did not occur in the case of *Gb’dac* rdRNAi (lower panel in Supplementary Fig. S1b; *n =* 12/13). These results suggest that *Gb’dac* is also essential for normal intercalary regeneration. This is consistent with the short-leg phenotype observed in tibia treated with rdRNAi against *Gb’dac*. It is interesting to note that similar phenotypes were observed in tibia treated with rdRNAi against *Gb’ds*/*Gb’ft^24,25^*.

**Supplementary Figure S1 | Effects of rdRNAi against *Gb’dac* on intercalary regeneration.**

(a) Schematic illustration of an intercalary regeneration experiment. The positional values (pvs) in the tibia (Ti) are denoted arbitrarily by the numbers 1 to 9. After amputation of a metathoracic leg (T3) at pv = 2 and a mesothoracic leg (T2) at pv = 8, a distal graft (g) of T2 is grafted to the proximal host stump (h) of T3. Intercalary regeneration occurs between pv 2 and pv 8, resulting in intercalation of the missing elements (pvs 3–7). (b, upper) A normally intercalated control tibia at the fifth instar. Bracket in control panel indicates the intercalated region. Rightward arrow indicates orientation of surface bristles. (b, lower) A regenerated tibia of a nymph treated with rdRNAi against *Gb’dac* at the fifth instar. No intercalary regeneration was observed. Fm, femur; Ti, tibia; Ta, tarsus. Scale bar in b = 200 µm.


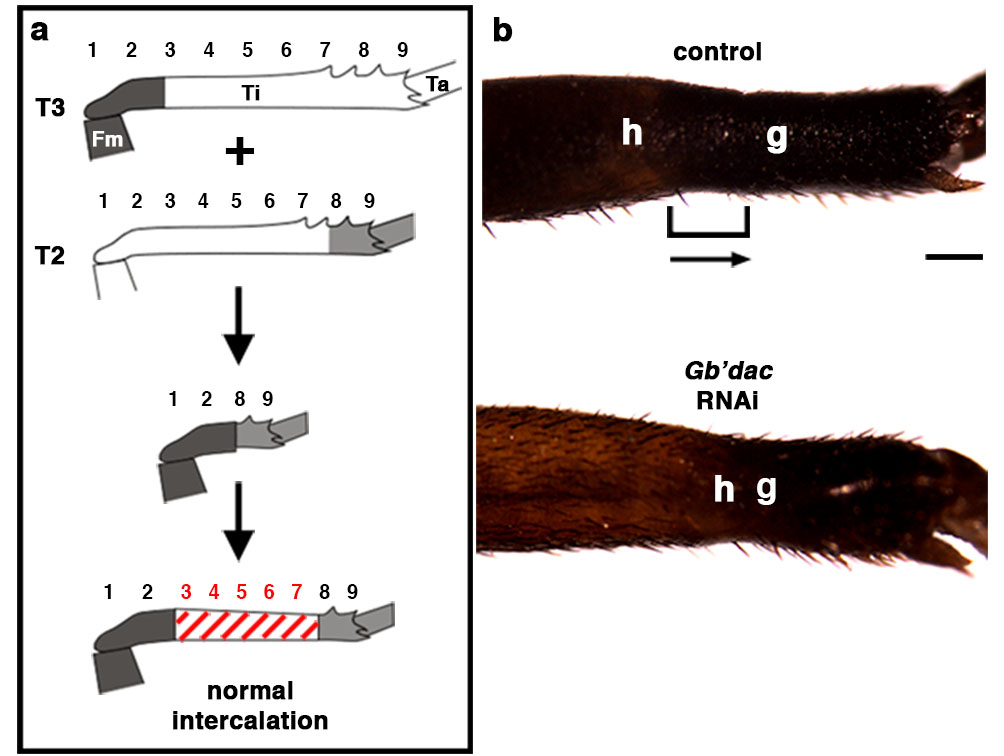


Supplementary Figure S1
